# Supplementary figures and images for: Transcriptome analysis and functional identification of GmMYB46 in soybean seedlings under salt stress
Source: PeerJ. 2021 Nov 11;9:e12492. doi: 10.7717/peerj.12492 (PMC8590805; doi:10.7717/peerj.12492)

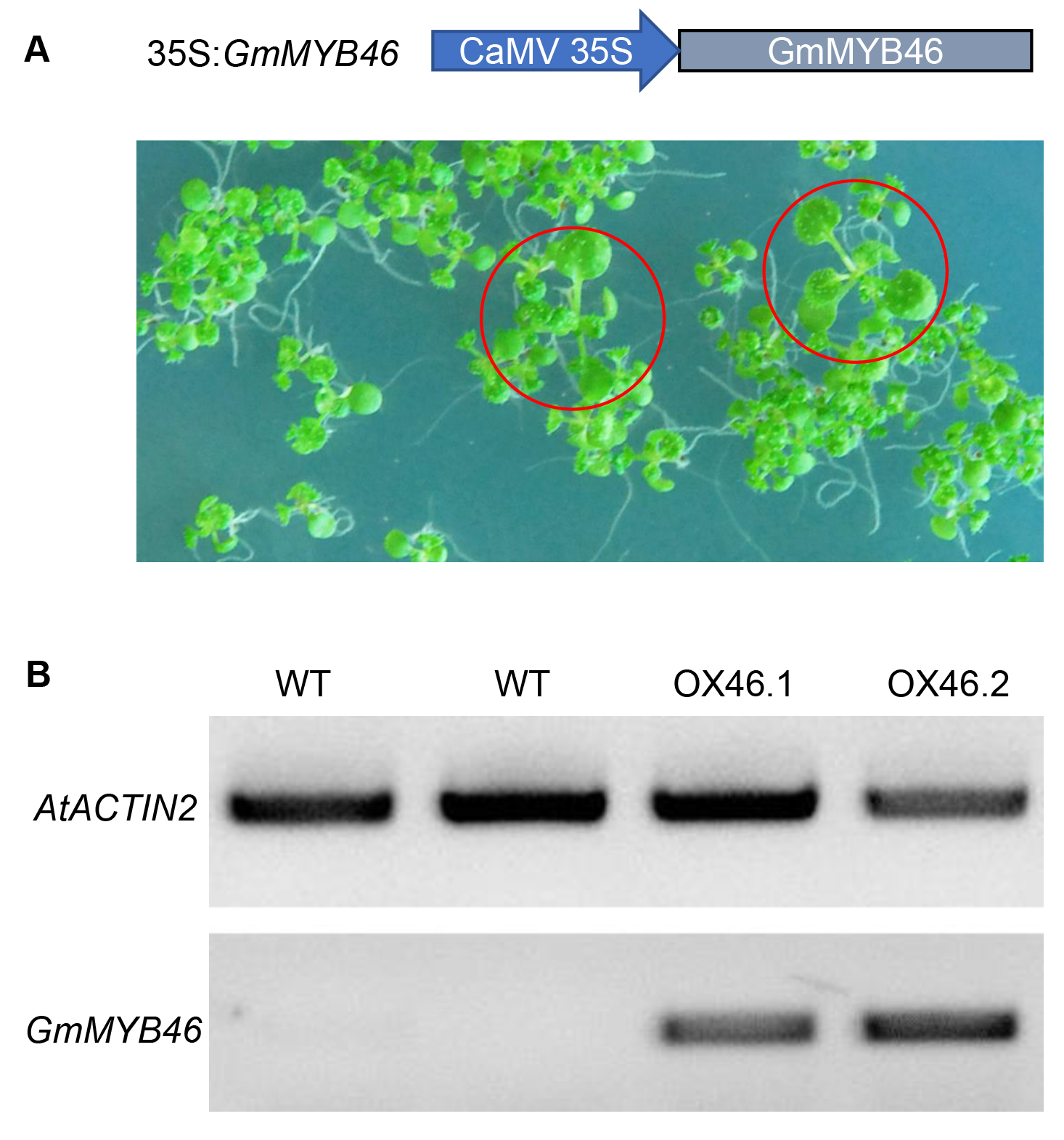

Supplement: Supplemental Information 1 — (A) Schematic diagram of overexpression vector construction and selection of hygromycin-resistant plants. (B) Expression identification of GmMYB46 in WT and overexpression lines. [file peerj-09-12492-s001.png]

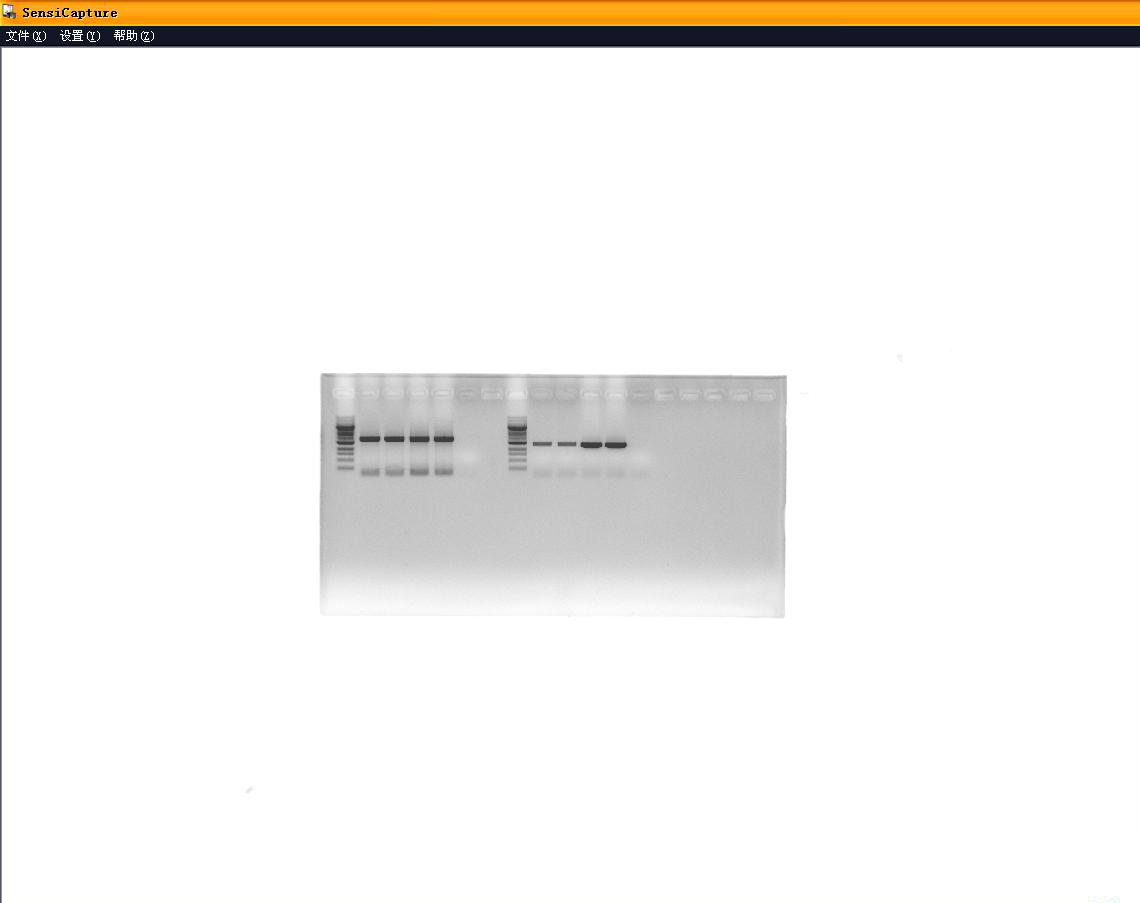

Supplement: Supplemental Information 11 [file peerj-09-12492-s011.zip › Uncropped blots for Figure 6C/Semi-quantitative PCR_1.jpg]

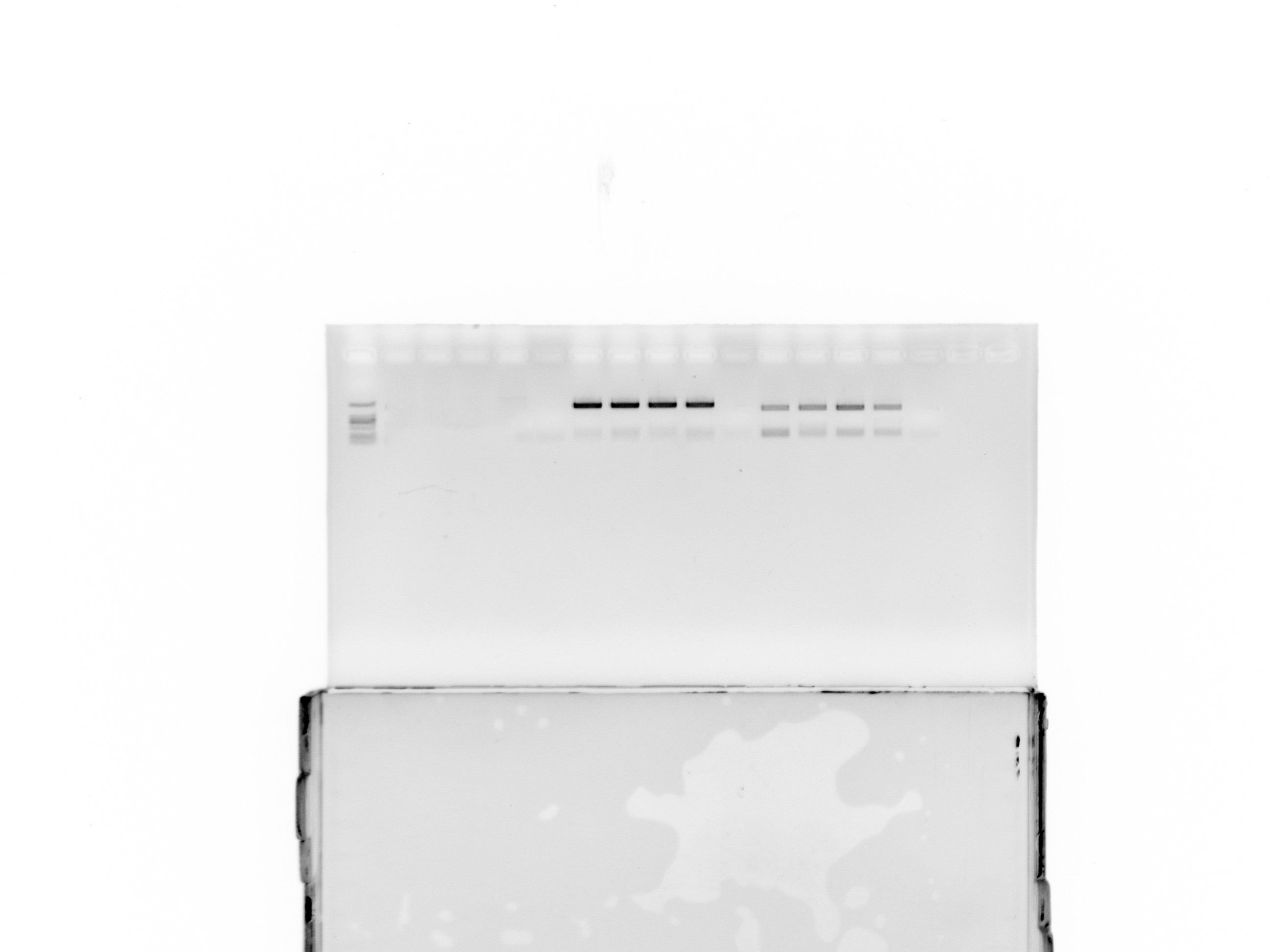

Supplement: Supplemental Information 11 [file peerj-09-12492-s011.zip › Uncropped blots for Figure 6C/Semi-quantitative PCR_2.jpg]
